# Neglected nontoxic multinodular goiter presented as a large neck mass

**DOI:** 10.1002/ccr3.771

**Published:** 2016-12-20

**Authors:** Stamatis S. Papadatos, Stefanos Mylonas, Christos Zissis, Vasiliki Galani

**Affiliations:** ^1^ 3^rd^ Department of Internal MedicineSotiria General HospitalNational and Kapodistrian University of Athens Medical SchoolAthensGreece; ^2^ 2^nd^ Department of Internal MedicineGeneral Hospital of TrikalaThessalyGreece; ^3^ Department of Anatomy‐Histology‐EmbryologyUniversity of Ioannina School of MedicineIoanninaGreece

**Keywords:** Iodine deficiency, multinodular goiter, neck mass

## Abstract

Large multinodular goiters, obvious to the naked eye, are rarely confronted by clinicians nowadays; yet they do have a place in the differential diagnosis of the neck masses. Due to the fact that 80% of the neck masses in adults are related to malignancy, the later should be ruled out.

A 69‐year‐old woman living in a village in Thessaly, Greece, was hospitalized for acute lower respiratory infection. She was found with a hard, painless, nodular yet asymptomatic neck mass. The trachea was midline and there was no intrathoracic extension of the mass. She reported that the mass had been gradually increasing for the last years, but she denied any compression symptoms and had not sought any medical advice. The patient was clinically and biochemically euthyroid. An ultrasound was ordered and revealed a grossly enlarged thyroid with multiple nodules >10 mm, the largest of which was 36 × 28 mm with a peripheral halo and absence of microcalcifications. No cervical lymphadenopathy was detected. The patient underwent a total thyroidectomy and the diagnosis of nontoxic multinodular goiter (NMG) was histopathologically confirmed. Given that the patient had never been exposed to any goitrogens, could you justify the origin of the NMG?

The incidence of either diffuse or nodular goiter is very much dependent on the iodine intake; in areas where iodine intake is insufficient, long‐standing and multinodular goiters are more frequently confronted. In the 1960s, several Greek areas were iodine deficient 1. In countries with previous deficiency corrected by salt iodination, elderly may have an incidence of 10% of nodular and multinodular goiter, due to lack of nutritional iodine in early life, compared with ~4% in countries without previous dietary iodine deficiency 2. A minority of those goiters are obvious to the naked eye. Large and complicated goiters may require medical and surgical treatment. In any case, the possibility of malignancy must be excluded (Fig. 1).

**Figure 1:**
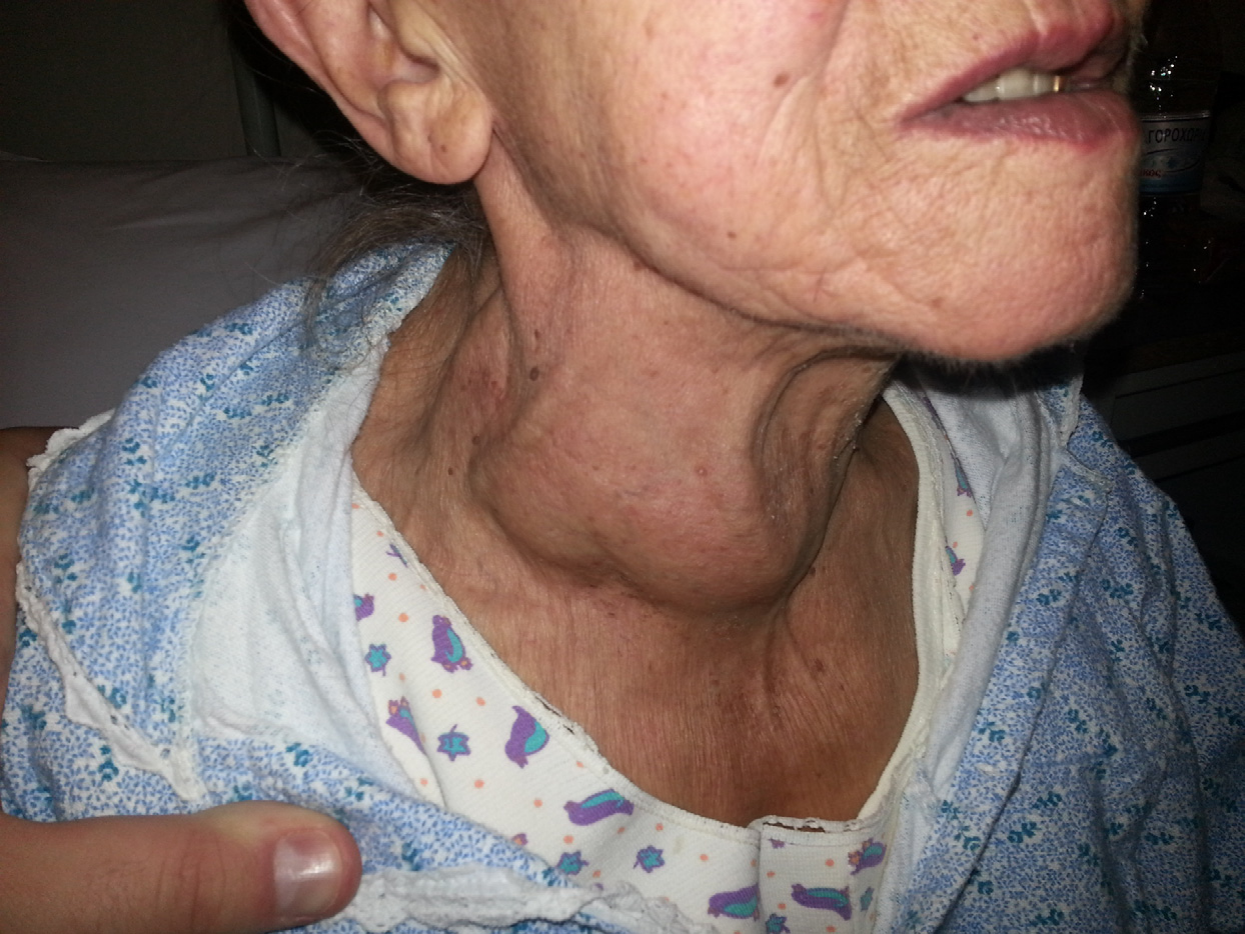
A large (euthyroid) multinodular goiter was evident in the neck. As iodine defficiency is not a prominent problen in Europe and North America nowadays, such clinical presentations are rarely met. Yet, there is high risk of thyroid cancer in patients with multinodular goiter, and therefore, the posibility of malignancy must be ruled out.

## Conflict of Interest

None declared.

## Authorship

SSP, SM, CZ, and VG: equally contributed to the conception of the work. SSP: wrote the first draft of the manuscript and all authors revised it critically and approved the final version to be published.
